# The Influence of Adding Spices to Reduced Sugar Foods on Overall Liking

**DOI:** 10.1111/1750-3841.14069

**Published:** 2018-02-24

**Authors:** John C. Peters, Ryan Marker, Zhaoxing Pan, Jeanne Anne Breen, James O. Hill

**Affiliations:** ^1^ Anschutz Health and Wellness Center Univ. of Colorado 12348 E. Montview Blvd. Mailstop C263 Aurora CO 80045 U.S.A; ^2^ Dept. of Pediatrics Univ. of Colorado Anschutz Medical Campus, 13123, E. 16th Ave., B065 Aurora CO 80045 U.S.A

**Keywords:** Flavor, liking, spices, sugar, sweetness

## Abstract

**Abstract:**

Reducing sugar intake is a major public health goal but many consumers are reluctant to use low calorie sweeteners. Two studies were conducted in healthy adults aged 18 to 65 to investigate whether addition of culinary spices to foods reduced in sugar could preserve hedonic liking. Test foods, black tea, oatmeal, and apple crisp, were prepared in full sugar (FS), reduced sugar (RS), and reduced sugar with spice (RSS) versions. Sugar reductions were 100%, 35%, and 37% for tea, oatmeal, and apple crisp, respectively. In Study 1, 160 subjects rated absolute liking of FS, RS, and RSS versions of a breakfast of oatmeal and tea and an afternoon snack of apple crisp on consecutive weeks. In Study 2, 150 subjects rated relative liking of all 3 versions of one food at the same seating, with different foods tested 1 wk apart. Liking was assessed using a 9‐point Likert scale. Both studies yielded similar results. For all 3 test items, liking was significantly higher for FS than for RS (*P* < 0.03). For tea, addition of spices did not significantly improve liking in either study. For oatmeal, addition of spices did not consistently improve liking compared to RS. For apple crisp, relative liking of RSS was not different then FS. These results indicate that it is possible to preserve the hedonic pleasure of a reduced sugar version of a dessert food, apple crisp, by addition of culinary spices. This may be a promising strategy to reduce sugar in some foods without using low calorie sweeteners.

**Practical Application:**

Reducing sugar consumption is an important public health goal. Many consumers are reluctant to use low calorie sweeteners and alternative approaches are needed. Using culinary spices to enhance the flavor of foods may allow sugar reduction while still preserving acceptable overall liking.

## Introduction

Added sugar in the diet has been identified as a significant contributor to the rise in obesity and chronic disease, both in the United States and globally (Malik, Schulze, & Hu, 2006; Malik et al., 2010; Johnson et al., 2007). To combat this issue, the U.S. Dept. of Health and Human Services and U.S. Dept. of Agriculture (2015) and the World Health Organization (2015) have recently released recommendations to limit added sugar to no more than 10% of total calories consumed. Adults in the United States currently obtain an average of 14.6% of calories from added sugars (Fitch & Keim, 2012; Wright & Wang, 2010). While reducing added sugars in dietary intake can be accomplished through the use of low calorie sweeteners (LCSs) such as aspartame, stevia, and sucralose (Fitch & Keim, 2012), many consumers are still wary of consuming these due to concerns about safety (Riobo Servan, Sierra Poyatos, & Soldo Rodriguez, 2014) and uncertain efficacy for helping with weight management (Fowler et al., 2008; Swithers, Martin, & Davidson, 2010). In addition, some consumers do not like the taste profile of these compounds when used in foods and beverages (Frank et al., 2008; Kamerud & Delwiche, 2007). Furthermore, there has been a general movement in the population to seek “natural” approaches to healthier eating (Falguera, Aliguer, & Falguera, 2012), which would avoid use of LCS as a means to reduce added sugars in the diet.

While the largest source of added sugars in the American diet is beverages (for example, soft drinks and sodas), accounting for approximately 33% of added sugars consumed, there is a substantial amount of added sugar consumed in other foods and beverages (Huth, Fulgoni, Keast, Park, & Auestad, 2013). Few studies, however, have investigated methods of reducing added sugars in foods and drinks other than carbonated beverages. Given the strong human desire for sweetness (Drewnowski, Mennella, Johnson, & Bellisle, 2012), the market viability of reduced sugar variations of food will depend, at least in part, on maintenance of the hedonic pleasure of the item. This, combined with the desire for “natural” approaches to healthy eating, raises the question as to whether there are alternatives to using LCSs that may reduce added sugar in foods and beverages while preserving sufficient hedonic pleasure to satisfy consumer liking.

We previously reported the successful use of herbs and spices to preserve the initial “liking” score of foods with reduced fat and saturated fat (Peters, Polsky, Stark, Zhaoxing, & Hill, 2014; Polsky et al., 2014). For foods like meatloaf, roasted chicken, sausage, French toast, and mixed vegetables, overall liking of reduced fat items with added herbs and spices was not different compared to full fat versions of those items, while preferring both of these options compared to plain reduced fat items. Thus, in single meal test situations, it was possible to substitute some portion of the flavor carried by dietary fat with herbs and spices without affecting overall food liking, suggesting a promising strategy for reducing calories and saturated fat in foods. While adding spices to reduced‐sugar items has been previously suggested (Fial, 1978), this concept has not been systematically investigated.

The present study examined the effects on overall liking of adding noncaloric culinary spices to RS versions of tea, oatmeal, and apple crisp as a potential strategy to preserve hedonic pleasure while reducing sugar intake.

## Materials and Methods

### Design

This study examined the overall consumer liking of different recipes of three food and beverage items using two different sequential monadic test designs: Study 1, participants tasted the three different versions of each test product 1 week apart (an indicator of absolute liking (AL)), and Study 2, participants tasted the three versions of each test product during the same session (an indicator of relative liking (RL)). Both studies were central location tests (participants came to the same location for all testing) and were single blind, randomized, 3‐period, within subjects, crossover designs. Participants were tested on the same day of the week, at the same time of day, and in the same place for each study. The three versions of each test item were (1) full sugar (FS), (2) reduced sugar (RS), and (3) reduced sugar with added spice (RSS). For each test item, the two RS versions contained the same amount of sugar and calories. The food and beverage items tested were tea, oatmeal, and apple crisp. In the 1st study (AL), these items were presented as a breakfast meal consisting of oatmeal and tea and an afternoon snack of apple crisp. In the 2nd study (RL), the three versions of each item were provided at a single tasting session, one version at a time, in randomized order within the session and according to a randomized weekly session sequence. In both studies, a Williams design (Wang, Wang, & Gong, 2009; Williams, 1949) was used so that the number of subjects receiving each of the 6 different possible treatment sequences was the same in order to balance for possible residual treatment effects. Subjects were blinded to the test item condition, although it was necessary that the staff preparing the items for service were not blinded to the test condition. The study was approved by the Colorado Multiple Institutional Review Board at the Univ. of Colorado, Anschutz Medical Campus. All subjects provided written informed consent. These trials were registered at clinicaltrials.gov (Study 1: NCT03134079; Study 2: NCT03139552).

### Study power, randomization, and treatment sequence

Limited data were available in the literature to estimate potential effect sizes for liking of foods with RS with and without spices. Therefore, study power was calculated based on the effect sizes observed in previous studies examining the effect on food liking of fat reduction with our without added herbs and spices (Peters et al., 2014). Based on the data from a similar study of food liking by Polsky and colleagues (Polsky et al., 2014), we assumed the between subject variance on the 9‐point Likert scale to be 0.29 and the within‐subject variance to be 1.3, which yielded a conservative estimate for the intraclass correlation coefficient (ICC) of 0.19. Furthermore, based on this previous work we assumed AL scores of 7, 6, and 6.9 for FS, RS, and RSS, respectively, with minor treatment sequence and period effects. A dropout rate of 20% was assumed based on our previous studies yielding a target enrollment of 150 subjects for each treatment condition in order to obtain 120 completers for each study. These sample sizes would provide greater than a 95% chance to detect statistical significance between FS and RS and between RS and RSS for 20 completer subjects for each of the 6 sequences tested in each study and using the mixed effects analysis model described later (Littell, Miliken, Stroup, Wolfinger, & Schabenberger, 2006).

Using a 3 × 6 Williams design (3 treatments, 6 possible treatment sequences) (Williams 1949), subjects were individually randomized into blocks of 6 participants, each with a different treatment sequence, representing all possible order combinations of the 3 test conditions (FS, RS, and RSS). A random list of 150 participants was generated using SAS procedure, Proc Plan.

### Subjects and screening

Adult participants between the ages of 18 and 65 were recruited from the Univ. of Colorado, Anschutz Medical Campus and surrounding areas using radio advertisements, public service announcements, notices in newspapers, campus announcements, and emails, flyers, and Craigslist or Facebook postings. Respondents were screened by telephone interview or by completion of an online questionnaire provided by email.

### Inclusion/exclusion criteria

Subjects meeting the age criteria were eligible if they were willing to sign an informed consent. Potential subjects were excluded if they reported diagnosed taste or sensory disorders that would prevent them from evaluating the food, pregnancy, known eating disorders, allergies to the test food/ingredients, medical conditions that may adversely affect taste (for example, dysgeusia), inability to complete the protocol, personal dietary restrictions toward test meal items (for example, gluten), and dislike of the particular food items to be served. Different subjects were recruited for the 2 studies. One hundred and sixty subjects were enrolled in Study 1 (AL) and 150 subjects were enrolled in study 2 (RL).

### Test food items

Recipes for the FS, RS, and RSS versions of tea, oatmeal, and apple crisp were the same for both studies. The test foods selected were designed to create 3 different added sugar scenarios: (1) an intrinsic sugar‐free item to which sugar is often added (tea), (2) an item with a small amount of intrinsic sugar (oatmeal with dried apples) to which sugar is often added, and (3) an item with considerable sugar from both intrinsic and added sugar (apple crisp with apples plus additional recipe sugar).

Tea recipes contained black tea (2.5 g/serving). The FS recipe contained sugar (2.2 g/serving), while the RS and RSS had no added sugar. The RSS tea also contained ground ginger (0.1 g/serving), cardamom pods (0.23 g/serving), and whole clove (0.13 g/serving). For study 1 (AL), the serving size was 240 mL (8 ounces). For study 2 (RL), 60 mL (2 ounces) was served because the participants would be tasting three different tea versions at one visit.

Oatmeal recipes consisted of quick oats (34.25 g/serving), dried apples (6.1 g/serving), and salt (0.45 g/serving). The RS and RSS recipes contained half the sugar of the FS recipe (14 g/serving compared with 7 g/serving, respectively). The RSS recipe also contained Saigon cinnamon (0.3 g/serving), Jamaican allspice (0.2 g/serving), and vanilla extract (0.1 g/serving). For both studies, the serving size was 166 to 173 g (about 5.5 ounces).

Apple crisp filling recipes contained apples (109.5 g/serving) and arrowroot (3.17 g/serving). The RS and RSS recipes had less added white sugar (2.9 g/serving) than the FS recipe (7.35 g/serving). The RSS apple crisp filling also contained Saigon cinnamon (0.43 g/serving). For both studies, serving size was between 147 and 158 g (about 5 ounces). Apple crisp topping recipes contained flour (7.98 g/serving), old‐fashioned oats (5.32 g/serving), salt (0.08 g/serving), and butter (10.39 g/serving). The FS apple crisp contained twice the amount of light brown sugar (7.34 compared to 3.67 g/serving) and white sugar (6.7 compared to 3.35 g/serving) as the RS and RSS recipes. The RSS apple crisp topping also contained twice the amount of Saigon cinnamon (0.35 compared to 0.17 g/serving) as FS and RS recipes.

Total calories and sugar contents for the test foods are shown in Table 1. The percent reduction in sugar for tea was 100%, for oatmeal 35%, and for apple crisp 37%.

**Table 1:** Total dietary sugar and calories by test meal condition

Meal item	Full sugar (FS)	Reduced sugar (RS, RSS)*	Reduction from full sugar
Tea			
Total calories (kcal)	10	0	100%
Total sugar (g)	2	0	100%
Oatmeal			
Total calories (kcal)	200	170	15%
Total sugar (g)	17	11	35%
Apple crisp			
Total calories (kcal)	270	230	15%
Total sugar (g)	30	19	37%

^*^Reduced sugar and reduced sugar plus spice.

### Study procedures

All study visits took place at the Anschutz Health and Wellness Center on the Univ. of Colorado Denver, Anschutz Medical Campus in Aurora, Colorado. Prior to taste testing participants completed an online version of the Block Food Frequency Questionnaire (FFQ, Version 2005) (Block, Woods, Potosky, & Clifford, 1990) designed to provide information about subjects’ demographics, body mass index (BMI), and typical dietary intake. Participants also completed a “Test Item Use Questionnaire” that specifically asked about subjects’ typical consumption patterns of the test items in the last year. For Study 2 (RL), this questionnaire was administered prior to testing, and for Study 1 (AL), this questionnaire was given retrospectively, approximately a year after subjects had already completed testing. These subjects also answered an additional query about whether they had made any significant changes to their usual consumption of the test items since participating in the taste test study. Of the 158 subjects contacted from Study 1 (AL), 126 subjects (79.7%) completed the Test Item Use Questionnaire.

Subjects arrived at Anschutz Health and Wellness Center 10 min prior to meal service and were checked in and assigned to individual tables and space such that test subjects could not converse with other subjects. They were given 12 ounces of room temperature water to drink with the test items and were not allowed to drink any other type of beverage. Participants were instructed to not talk to other participants, read or talk on the phone during the meal to ensure they could focus on their evaluation of each item. They were told to not consume any food or drink besides water for 2 hr prior to their scheduled tastings. Subjects were served their test meal/item for that visit according to a randomized and balanced treatment sequence schedule.

### Test food item evaluation

Food liking in both studies was evaluated using a 9‐point hedonic scale (Likert) anchored at one end by “dislike extremely” and at the other by “like extremely.” Pictures of the individual test food items were taken before and after completion of the tasting in order to confirm that each food item was tasted. Individual items were weighed before being served to subjects and again after testing and the weight of food consumed was calculated as an indicator of how liking affected amount of food consumed.

Study 1 (AL): Participants were asked to rate liking of the three different versions of tea, oatmeal, and apple crisp on successive weeks. Oatmeal and tea were served at the same seating with participants choosing a seating time of either 7 AM, 8 AM, or 9 AM. Apple crisp was served at 2 PM, 3 PM, or 4 PM. Participants were given 30 min to eat the test items and complete the rating form. Each seating accommodated up to 12 subjects allowing up to 36 subjects to be served on each test day. Individuals were tested on the same day of the week and same hour of the day for each of the three test meals.

Study 2 (RL): Participants were asked to rate liking of the FS, RS, and RSS versions of 1 food item at the same seating on successive weeks. They were asked to take at least four bites and/or sips of each item but were told that they did not have to finish any item. Portion sizes for oatmeal and apple crisp were the same as those in Study 1 (AL), while the portion sizes for tea in Study 2 (RL) were reduced from 8 ounces to 2 ounces, given that subjects were asked to taste all three at one seating. Each version of a test item was presented by itself and was evaluated, then the test item was taken away and the next version of the item was presented and evaluated such that only 1 test item was available to the subjects at any time. Participants were instructed to take a few bites and sips of unsalted cracker and water, respectively, in between tasting each test item in order to cleanse their palate. Thirty minutes were allowed to taste the items and complete the evaluation questionnaire. After evaluating the liking of all 3 items, participants rank ordered their liking of the items, 1st, 2nd, and 3rd best liked.

### Statistical analysis

Statistical analyses were performed using SAS (SAS Inst., Cary, N.C., U.S.A.). For both Studies 1 and 2, a linear mixed effects model was created for each of the 3 food items to evaluate differences in liking scores between the FS, RS, and RSS conditions. These models consisted of condition (FS, RS, and RSS), sequence (of condition), and period (for Study 1, Week 1, Week 2, Week 3; for Study 2, time was not a factor) as fixed effect predictors and a random subject effect. Least square estimates of differences between conditions were tested and the 95% confidence interval for these differences was estimated under the model. These same analyses were repeated to investigate differences in the percent consumed for each condition. In Study 2 (RL), for the ranking data, a chi‐square or Fisher's exact test, as appropriate, was used to determine which version of each test item was ranked first among the FS, RS, and RSS versions.

The test item use questionnaire was used to characterize the study population relative to their frequency of use of each of the test foods over the previous year. In order to determine whether the familiarity with the specific test item might have affected liking, *t*‐tests were used to compare liking scores of food item conditions between frequent and infrequent consumers of the respective food item. Significance for all statistical analyses was defined as *P* < 0.05.

The distribution of liking scores for the test items was not normally distributed and was slightly skewed toward higher median ratings. Because of this, nonparametric analyses (Wilcoxon rank sum test) were also conducted which produced the same results as parametric methods. This skewness should not pose problems with the primary analyses using mixed effect modeling since the sample sizes are large. All results presented are for the parametric tests.

## Results and Discussion

Characteristics of the study population for Studies 1 (*N* = 160) and 2 (*N* = 150), including consumption frequency categorization for the test food items, are shown in Table 2 and 3. For both studies, the test populations were predominately female, Caucasian, and non‐Hispanic with average ages ranging from 33 to 38 years and with BMIs averaging 25. Typical dietary intakes of total calories, protein, carbohydrate, and fat based on the Block food frequency questionnaire were similar across studies and were similar to the U.S. population as a whole (Austin, Ogden, & Hill, 2011). Reported intakes of added sugar, sugar sweetened beverages, and sweet desserts were similar between the two studies. It is recognized that food frequency instruments do not provide an accurate quantitative assessment of consumption amount or caloric intake; however, they can be used to estimate relative dietary quality (Block et al., 1990; Subar et al., 2001). Data from the two studies indicate that the overall dietary patterns of the test populations were qualitatively similar to one another and were similar to the U.S. adult population as a whole.

**Table 2:** Study 1: Subject characteristics, habitual diet, and consumption frequency of select food items (*N* = 160)

Characteristic		Result
Sex, *n* (%)		
	Women	132 (82.5)
	Men	28 (17.5)
Age, years (range)		38.1 (23–65)
Race, *n* (%)		
	Caucasian	143 (89.4)
	African American	4 (2.5)
	Asian	8 (5.0)
	Other	5 (3.1)
Ethnicity, *n* (%)^a^		
	Non‐Hispanic/Latino	131 (82.4)
	Hispanic/Latino	18 (11.3)
	Other	10 (6.3)
BMI (kg/m^2^), mean (SD)		24.8 (5.3)
Total calories per day, mean (SD)^a^		1614.4 (672.5)
Percentage of kcals from:	Percent from fat	37.8 (5.7)
	Percent from protein	15.6 (2.6)
	Percent from carbohydrate	45.7 (6.7)
	Percent from sweets/desserts	10.6 (7.2)
Added sugars per day, g^a^		36.5
Apple crisp consumption, *n* (%)^b^		
	Frequent^c^	2 (1.6)
	Infrequent^d^	125 (98.4)
Oatmeal consumption, *n* (%)^b^		
	Frequent	34 (26.8)
	Infrequent	93 (73.2)
Hot tea consumption, *n* (%)^b^		
	Frequent	67 (52.8)
	Infrequent	60 (47.2)

BMI = body mass index.

^a^Data missing from one participant (*N* = 159).

^b^Data collected retrospectively approximately 15 mo after taste testing on 127 subjects (*N* = 127).

^c^Frequent defined as consumed a few times per week, more days than not, or daily in last year.

^d^Infrequent defined as consumed occasionally, seldom, or never in last year.

**Table 3:** Study 2: Subject characteristics, habitual diet, and consumption frequency of select food items (*N* = 150)

Characteristic		Result
Sex, *n* (%)		
	Women	118 (78.7)
	Men	32 (21.3)
Age, years (range)		32.9 (21–65)
Race, *n* (%)^a^		
	Caucasian	119 (80.4)
	African American	7 (4.7)
	Asian	18 (12.2)
	Other	4 (2.7)
Ethnicity, *n* (%)^b^		
	Non‐Hispanic/Latino	114 (78.6)
	Hispanic/Latino	16 (11.0)
	Other	15 (10.3)
BMI (kg/m^2^), mean (SD)		25.0 (4.5)
Total calories per day, mean (SD)		1616.7 (528.3)
Percentage of kcals from:	Percent from fat	38.1 (5.7)
	Percent from protein	15.5 (2.8)
	Percent from carbohydrate	44.9 (7.0)
	Percent from sweets/desserts	10.9 (6.7)
Added sugars per day, g		34.4
Apple crisp consumption, *n* (%)^a^		
	Frequent^c^	2 (1.4)
	Infrequent^d^	146 (98.6)
Oatmeal consumption, *n* (%)^a^		
	Frequent	46 (31.1)
	Infrequent	102 (68.9)
Hot tea consumption, *n* (%)^a^		
	Frequent	74 (50.0)
	Infrequent	74 (50.0)

BMI = body mass index.

^a^Data missing from 2 participants (*N* = 148).

^b^Data missing from 5 participants (*N* = 145).

^c^Frequent defined as consumed a few times per week, more days than not, or daily in last year.

^d^Infrequent defined as consumed occasionally, seldom, or never in last year.

Based on the Test Item Use Questionnaire (see Table 2 and 3), approximately half of the test population in both studies consumed hot tea on a frequent basis (defined as consumed a few times per week, more days than not, or daily in last year) and half as infrequent consumers (occasionally, seldom, or never in last year). Habitual oatmeal consumption was similar between the studies and ranged from 27% to 31% of frequent consumers. Apple crisp consumption was also similar between studies but only 2% of the participants reported frequent consumption. Despite the low frequency of apple crisp consumption, intake of sweet desserts as a category, of which apple crisp is one example, was much greater and represented nearly 11% of daily calorie intake based on the Block FFQ data (Table 2 and 3). Overall, the test foods chosen are representative of typical foods eaten by the U.S. population.

### Overall liking, amount consumed, and item ranking

**Figure 1:**
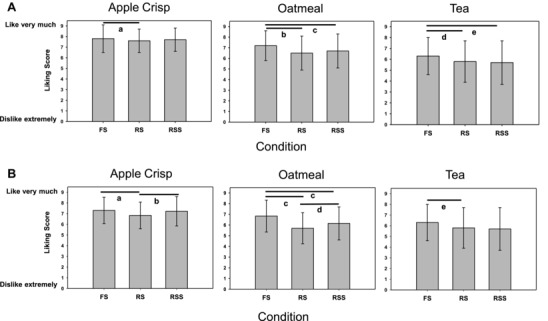
(a) Study 1 (absolute liking study), overall liking scores on 9‐point Likert scale (mean ± SD). *N* = 141, 144, and 143 for FS, RS, and RSS, respectively. **^a^** *P* = 0.012, **^b^** *P* < 0.0001, **^c^** *P* = 0.003, **^d^** *P* = 0.008, **^e^** *P* = 0.0008. (b) Study 2 (relative liking study), overall liking scores on 9‐point Likert scale. *N* = 150; **^a^** *P* = 0.0003, **^b^** *P* < 0.0025, **^c^** *P* = 0.0001, **^d^** *P* = 0.003, **^e^** *P* = 0.03.

Overall liking scores for the different test food items and conditions (FS, RS, and RSS) for Study 1 (AL) and Study 2 (RL) are shown in Figure 1(a) and (b), respectively. In both Studies 1 and 2, compared to FS, reducing sugar in tea significantly reduced liking and the addition of spices did not restore mean liking. There was no difference in liking between RS and RSS in either study. Similar results were seen for oatmeal. Compared to FS, both RS and RSS versions were liked significantly less. Addition of spices increased mean liking slightly compared to the RS, but the increase was significant only in Study 2, and RSS was still liked significantly less than FS. For apple crisp, in both studies, liking of the RS version was significantly less than FS, while liking of RSS was not different from FS (see Figures 1a and b). In Study 2, liking of the RSS version (rating 7.2) was significantly greater than for RS (rating 6.8). Although there were statistically significant differences between the different versions of apple crisp, liking was high for all three versions.

**Figure 2:**
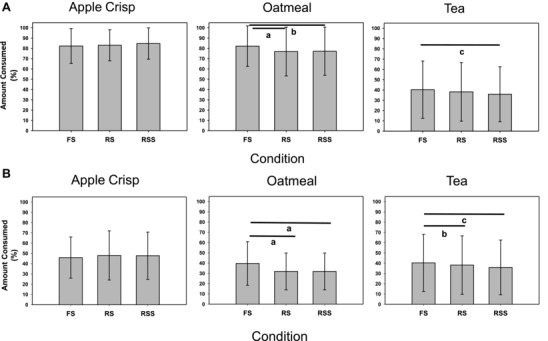
(a) Study 1 (absolute liking study), amount consumed (%). *N* = 141, 144, and 142 for FS, RS, and RSS, respectively. **^a^** *P* = 0.003, **^b^** *P* = 0.005, **^c^** *P* < 0.01. (b) Study 2 (relative liking study), amount consumed (%) (mean ± SD). *N* = 150; **^a^** *P* < 0.0001, **^b^** *P* = 0.013, **^c^** *P* = 0.04.

The amount of each food item consumed for Studies 1 and 2, is shown in Figure 2(a) and (b), respectively. In general, the results for amount consumed followed a pattern similar to those for liking of each food item. Because all three versions of each test food were sampled at the same seating in Study 2 (RL), the absolute amounts eaten were less than in Study 1 (AL). For tea, in Study 1 (AL), compared to FS, there was less consumption of both RS and RSS and the difference for RSS was significant. In Study 2 (RL), both RS and RSS were consumed in significantly lesser amounts than FS. For oatmeal, in both Studies 1 and 2, participants consumed significantly less of the RS and RSS versions compared to FS. For apple crisp, within both studies, there were no significant differences between versions in the amount consumed.

In Study 2 (RL), participants ranked which version of each test food item they liked the best (Table 4). For tea and oatmeal, more participants ranked the FS version as their 1st choice and the RS version least preferred among the 3 recipes. For apple crisp, more people ranked the RSS version highest compared to either FS or RS.

**Table 4:** Percent of participants ranking a given test item as their best‐liked version (compare ranking distributions vertically for each column).^a^

	Tea (%)	Oatmeal (%)	Apple crisp (%)
Full Sugar (FS)	44	60	36
Reduced Sugar (RS)	23	8	22
Reduced Sugar Spice (RSS)	33	32	42

^a^Data from Study 2 in which relative ranking was assessed.

### Frequent compared with infrequent consumption

For both studies, either individually or combined, there were no statistically significant differences in liking ratings of oatmeal or apple crisp whether participants were frequent or infrequent consumers of those items in their regular diet. However, these were secondary analyses and the study was not powered to detect small differences in liking ratings based on frequency of use. Combined results (Studies 1 and 2) for tea, however, showed that frequent consumers of tea rated RS tea higher than did infrequent consumers (5.9 compared with 5.4 rating, on the 0 to 9 Likert scale; *P* = 0.032). In addition, for those participants who reported infrequent habitual use of sweetener in their tea, ratings for the RS and RSS versions of the tea were higher compared to participants who reported frequent use of sweetener in their tea (*P* = 0.022, *P* = 0.002, for RS and RSS, respectively). Frequent tea consumers also rated RSS tea higher than infrequent consumers, although the result did not reach significance (6.0 compared with 5.6 rating; *P* = 0.088). Liking of RS and RSS teas was significantly greater for frequent tea consumers who did not habitually used sweetener in their tea compared to those who typically use sweeteners (*P* = 0.048, *P* = 0.028, for RS and RSS respectively). Despite this, the AL ratings of RS and RSS tea among frequent tea consumers were still lower than that of FS tea (6.13 rating), and among nonsweetener users. It is possible that frequent consumers of tea prefer less sweet tea compared to infrequent consumers perhaps because they like the herbal/spice flavor as the main flavor contributor rather than sweetness. It is also possible that individual differences in sensitivity to bitter taste (for example, flavor constituents in tea) may influence frequency of tea consumption as well as preference for sweetened tea, although there is no conclusive evidence that bitter taste sensitivity (for example, as assessed by 6‐n‐Propylthiouracil taste) is predictive of consumption of bitter foods or beverages (Duffy & Bartoshuk, 2000; Tepper, 1998).

These studies showed that it is possible to reduce the amount of sugar in a high sugar food and still preserve liking, at least in the short term, by adding culinary spices to enhance the overall flavor profile. When the level of sugar in apple crisp was reduced by 37% and the flavor was augmented by the addition of spices, overall liking and the amount consumed were not different from the FS version. In fact, in Study 2 (RL), when the 3 versions of each food were rated for preference, more participants rated the RSS apple crisp as their number one preference compared to the FS version. All 3 versions of apple crisp were well liked, and although statistically significant differences were observed between the FS and RS versions and the RS and RSS versions for liking, the absolute differences in rating were small and may not be of practical significance. However, the observation that sugar could be reduced by 37% from a standard recipe without significantly reducing liking is a promising result. Furthermore, the finding that adding spices to the RS version of apple crisp fully preserved liking relative to the FS version and that the RSS version was ranked number one by the most participants supports the conclusion that it is possible to reduce sugar in some foods through the use of culinary spices without sacrificing hedonic liking.

In contrast to the results seen with apple crisp, overall liking was not fully restored to that of FS when spices were added to RS versions of oatmeal or black tea. In the case of oatmeal, this may have been due to the relatively limited intensity and complexity of the flavor display in the RS version, having only oats and dried apples as major flavor elements. Adding sugar (or other sweetener), which is the most commonly added ingredient by our participants in typical use, increased both the intensity and profile of flavor and was associated with improved liking. Adding cinnamon alone to the RS recipe was not able to increase the flavor experience sufficiently enough to restore liking to the level of the FS condition. Tea was a much more difficult flavor matrix to work with and its ratings may have suffered because it was difficult to prepare a test version that adequately represented the many different individual participant preferences for the type of tea (black, green, herbal, and so on) and the method of preparation (for example tea strength, adding milk), which significantly affect the overall flavor profile and intensity. Because tea already constitutes a kind of spice, it may be difficult to further intensify the total spice flavor without disturbing liking. AL scores for FS, RS, and RSS tea were lower than for the other food items tested indicating that the specific tea preparation tested was not well liked by most of the participants. Despite this, they were still able to provide significantly different ratings for the different versions of tea presented.

Overall, these results suggest that reducing sugar in the diet may be difficult and the use of spices to accomplish this may depend on the types of foods targeted. Future research should investigate the boundary conditions for RS food contexts within which spice addition may be effective in satisfying hedonic liking. The present results suggest that multiple‐ingredient foods high in total sugar may be the most promising targets for sugar reduction. One could hypothesize that a threshold amount of sugar may be sufficient to satisfy the preference for sweetness and that total flavor, and hence overall liking, is also a function of the flavors provided by other ingredients, including culinary spices. Adding more sugar to a recipe may affect total flavor and overall liking in one‐dimensional fashion by simply increasing sweetness. It has been shown that sweetness perception increases in linear fashion while sweetness pleasantness does not and after reaching an optimum, declines (Moskowitz, Kluter, Westerling, & Jacobs, 1974). Using apple crisp as a hypothetical model food example, it is possible that once some minimal “sweetness preference threshold” is met, overall liking may be driven by other recipe ingredients contributing to total flavor, including fat, apples, baked grain, and added culinary spices. Foods like tea and oatmeal that are lower in sugar to start with and have fewer flavor‐contributing elements may be more dependent on sugar as a major contributor to total flavor. Reducing sugar in these foods may have a more detrimental effect on overall liking, which cannot be restored by simply adding more flavor from spices as the “sweetness threshold” may not have been met.

Few studies have systematically examined sweet preference thresholds in different foods. Wise, Nattress, Flammer, and Beauchamp (2016) looked at whether sweet preference and sugar intake could be reduced by habituating subjects to a reduced sugar diet. During the 3‐mo intervention with a low sugar diet subjects indeed reported that test foods like pudding and a sweet beverage tasted sweeter than prior to the intervention, suggesting that sugar may behave like salt in that habituation to a low salt diet reduces future preference for and intake of salt (Beauchamp, Bertino, & Engelman 1983; Beauchamp, Bertino, & Engelman 1987; Bertino, Beauchamp, & Engelman, 1982). However, this was not the case for sugar. While foods were rated as sweeter at a given sugar concentration after habituation to reduced sugar intake than before habituation, when subjects were then allowed to select their own diet they still preferred the same (higher) level of sweetness as prior to the experiment. This suggests that subjects may not have maintained a reduced sugar intake longer term. Whether or not it is possible to habituate a preference for reduced sugar or sweetness among individuals habituated to a high sugar/sweet diet remains to be explored further in long‐term human studies.

Our previous work with fat reduction showed that it was possible to remove substantial fat and saturated fat in a variety of foods yet maintain overall liking at a single meal occasion through the addition of various herbs and spices to the recipe. While taste receptors for fat (fatty acids) have been described (Besnard, Passilly‐Degrace, & Khan, 2016; Pittman, 2010), fat may differ from sugar in that it also serves as a flavor carrier and contributes to the mouthfeel of foods. Addition of herbs and spices to reduced fat foods may help maintain an overall flavor intensity despite reduction of fat‐associated flavor. However, we found that this strategy did not work when the role of fat in the specific food involved producing a creamy mouthfeel such as pasta Alfredo (Peters et al., 2014).

The present data suggest it may not be possible to preserve overall liking of reduced sugar foods by adding spices when sweetness, *per se*, is a prominent feature of the flavor experience for foods with low to moderate intrinsic sugar and sweetness levels, as for tea and oatmeal in the present study. For foods with high levels of sugar and sweetness, and a more complex flavor profile, it may be possible to reduce the sugar level while maintaining a high level of flavor through the addition of spices. Apple crisp may represent one such example of a food whose liking may be preserved after sugar reduction by the addition of spices. It may also be that the specific spices added to the apple crisp, like vanilla, actually contributed some sweet character independent of the sugar. One study showed that vanilla was rated as the most sweet spice among a test battery (Blank & Mattes, 1990), and indeed, vanilla was one of the spices used in the RS apple crisp used in the present study. However, we also found that the RS apple crisp without additional spices was highly rated by participants. This may suggest that there may be a threshold level of sweetness in apple crisp that satisfies a hedonic preference and adding more sugar does not further enhance liking. Adding spices to this reduced sugar item did increase liking, suggesting that spices may have enhanced the overall flavor profile of the food. Finally, while spices may have limited ability to work broadly across foods as a strategy to reduce sugar, when combined with other approaches using herbs and spices to reduce salt (Anderson et al., 2015 and fat intake (Peters et al., 2014; Polsky et al., 2014), they may be useful adjuncts to public health strategies aimed at improving diet quality.

The strengths of this study include using 2 different test paradigms, one for AL (Study 1) and one for RL (Study 2), and the studies had relatively large sample sizes. Both studies produced similar findings. In addition, we tested typical foods having a range of different intrinsic sugar levels. Studies using real foods have a number of limitations, however, including the variation in response introduced by individual subject differences in experiences and expectations with the test foods based on how they might typically prepare and consume them. Other limitations include the larger number of women in the test sample compared to men. Finally, we did not have a FS control that also included added spices, which may have increased overall liking and further widened the liking gap with the RS and RSS versions.

## Conclusions

These studies show that it is possible to substantially reduce the sugar in a high‐sugar, flavor‐complex dessert food while still preserving hedonic liking through the addition of culinary spices. Using spices to preserve liking in foods where sweetness is the major contributor to flavor may be more difficult. Despite this, our findings are promising and suggest that culinary spices may be a useful alternative to LCSs as a strategy to reduce sugar in some foods. Even a small impact of spices on sugar intake, when combined with the utility of culinary herbs and spices to reduce fat and salt, could be helpful in promoting healthy eating.

## Author Disclosures

Funded by an unrestricted research gift from the McCormick Science Institute. Dr. Hill serves on the scientific advisory board for the McCormick Science Institute.

## Author Contributions

J. Peters, Z. Pan, and J. Hill were responsible for study design and outcomes analyses. J. Breen was responsible for scheduling and conducting study visits, collecting and entering study data for analysis, and helped design the tables. Z. Pan analyzed the data for the manuscript. R. Marker assisted in data interpretation and wrote an early draft of Study 1. R. Stark scheduled study participants, conducted study visits, collected data, and entered study data for analyses. Z. Pan analyzed the data for the manuscript. J. Peters was responsible for data interpretation and writing the manuscript. J. Hill reviewed and helped edit the manuscript.

**Nomenclature**
FS =: Full sugar
RS =: Reduced sugar with no added spice
RSS =: Reduced sugar plus spice
